# Dr. Asa S. Couch

**Published:** 1917-03

**Authors:** 


**Dr. Asa S. Couch,** Hahnemann of Philadelphia 1855, died in N. Y. City, Feb. 1, aged 81. The remains were interred at Fredonia, his late home, with Masonic honors.

